# Disulfide bonds stabilize the SARS-CoV-2 RBD and preserve binding to ACE2 following thermal denaturation

**DOI:** 10.1042/BSR20260440

**Published:** 2026-09-29

**Authors:** Nathan R. McCann, Francis J. Castellino

**Affiliations:** The W.M. Keck Center for Transgene Research and the Department of Chemistry and Biochemistry, The University of Notre Dame, Notre Dame, IN 46556, U.S.A.

**Keywords:** binding kinetics, binding thermodynamics, protein folding, protein stability, receptor-binding domain (RBD), SARS-CoV-2

## Abstract

The Spike protein of severe acute respiratory syndrome coronavirus 2 (SARS-CoV-2) mediates host cell attachment and entry through the binding of its cognate receptor, angiotensin-converting enzyme 2 (ACE2). This interaction occurs within the receptor-binding domain (RBD) of the Spike protein and is critical to the lifecycle and fitness of SARS-CoV-2. A rigorous analysis of this interaction is crucial to understanding this viral entry mechanism. In the present communication, a biophysical examination of the RBD, ACE2, and their interaction is presented. These studies led to the discovery that disulfide bonds stabilize and protect the RBD from thermal and chemical denaturation. It is further hypothesized that disulfide bond stabilization preserves the ability of the RBD to bind ACE2 following its denaturation. The present hypothesis is supported by the finding that the RBD retains ACE2-binding activity after treatment to high temperatures (95°C) under non-reducing, but not reducing, conditions. These results suggest a mechanism by which SARS-CoV-2 overcomes environmental stress to preserve its RBD–ACE2 interaction.

## Introduction

Severe acute respiratory syndrome coronavirus 2 (SARS-CoV-2) [1–3] was the etiological agent behind the coronavirus disease 2019 pandemic [4]. SARS-CoV-2 utilizes its Spike protein [5–8], a trimeric class I viral fusion protein [9], to initiate infection through the binding of its host cell receptor, angiotensin-converting enzyme 2 (ACE2) [10–12]. This high-affinity, multivalent interaction enables the stable attachment of SARS-CoV-2 to the host cell. Following receptor binding, Spike undergoes a drastic conformational transition from a pre-fusion to a post-fusion state [13] upon proteolytic activation by transmembrane protease serine 2 at the cell surface [11,14–16] or Cathepsin L in the endosome [11,17]. This conformational change facilitates the fusion of viral and host cell membranes, which culminates in the release of the viral genome into the host cell and the renewal of the SARS-CoV-2 lifecycle [18].

The Spike protein consists of three identical protomers, which are 1273 amino acids in length and are composed of several domains and subunits that are critical to its function. Spike also contains 22 N-linked glycans within each protomer [19–21] that influence its folding [22], receptor binding [23–26], and antibody evasion [26–29] abilities. The S1 and S2 subunits of Spike are responsible for receptor binding and membrane fusion, respectively. Spike interacts with ACE2 through its receptor-binding domain (RBD), which is located within the S1 subunit and spans amino acid residues 331–528. The RBD is composed of five antiparallel β-sheets that are connected through random loops and α-helices [30]. It also features four disulfide bonds between residues C336–C361, C379–C432, C391–C525, and C480–C488 (Figure 1A,B). The physical contacts between the RBD and ACE2 occur within the receptor-binding motif (RBM) of the RBD. The RBM is located within amino acid residues 438–506 and is an unstructured loop with a concave shape. This structure cradles the N-terminal helix-turn-helix motif of ACE2 and forms a stable interaction with the receptor. Each RBD within Spike alternates between up and down states. As shown by cryo-EM reconstructions, the up state is solvent exposed and capable of binding ACE2, while the down state conceals the RBM, thereby preventing this interaction [5–8]. Since the RBD is the primary target of neutralizing antibodies directed toward Spike [31–36], it has been hypothesized that its conformational equilibrium plays a role in evading host antibody defenses [7,37–39]. In agreement with this perspective, Spike proteins engineered with all RBDs locked in the down state have been demonstrated to avoid binding antibodies directed toward the RBD and reduce serum neutralization [40,41].

**Figure 1:**
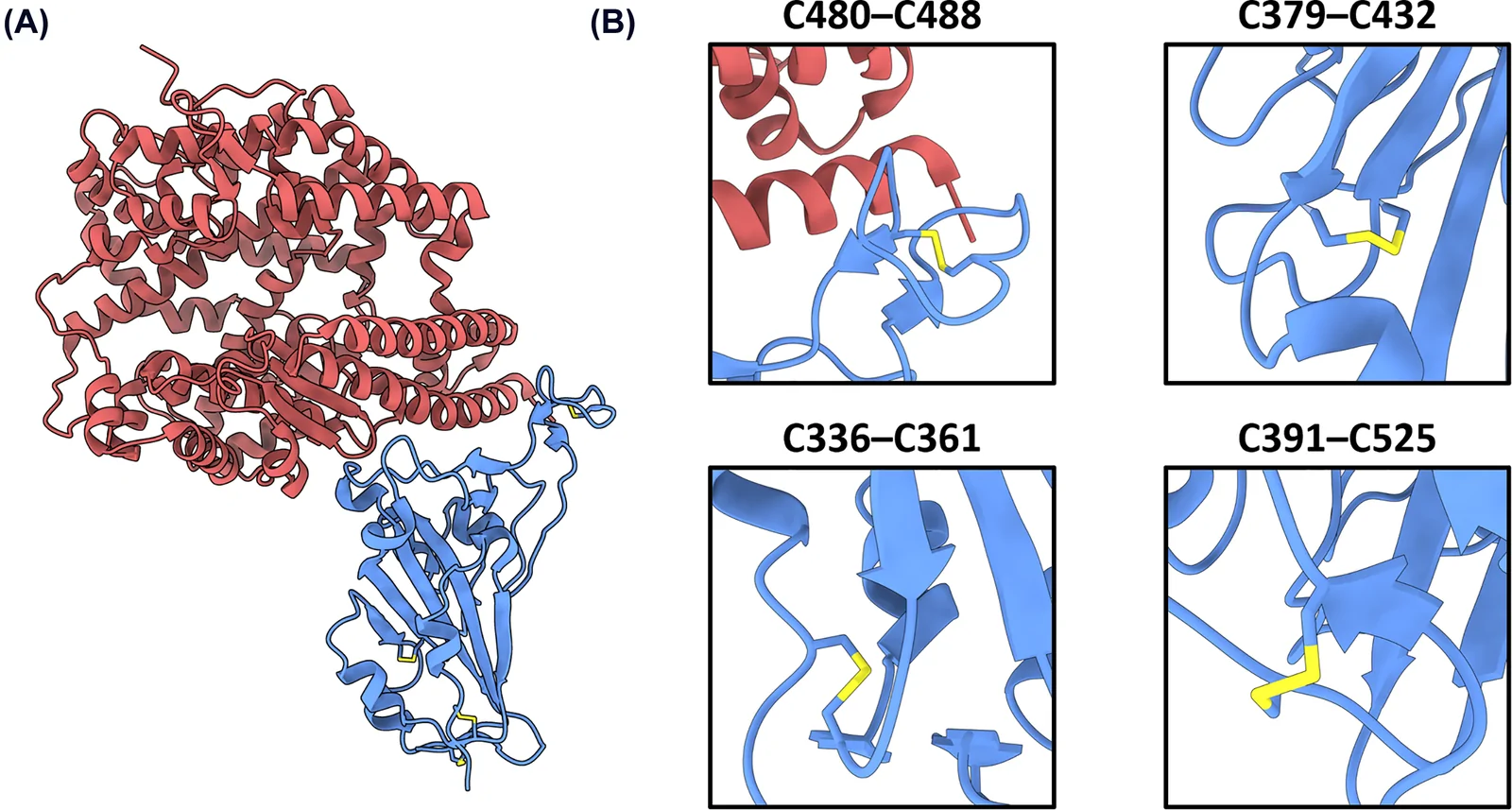
Structural impact of SARS-CoV-2 RBD disulfide bonds (**A**) Structure of the SARS-CoV-2 RBD bound to ACE2 (PDB ID: 6M0J) [30]. (**B**) Zoomed-in images of disulfide bonds within the RBD.

Although the utilization of ACE2 as the host cell receptor is a conserved trait among many SARS-related viruses [11,12,42–49], the recognition and binding of human ACE2 represent an important barrier to their zoonotic transmission [12,50]. Following the spillover of SARS-CoV-2 into humans, the virus rapidly evolved to adapt to its new host. SARS-CoV-2 variants emerged with enhanced receptor affinities [51–57] and abilities to evade host antibody defenses [58–81]. These properties emerged from substitutions acquired by the Spike protein, many of which were located in the RBD.

Receptor binding is the first and most critical step in the entry of SARS-CoV-2 into host cells. The stable attachment of the virus to the cell surface is required for downstream infection. Therefore, a thorough understanding of the RBD–ACE2 interaction is crucial to prepare for the emergence of future SARS-CoV-2 variants and the possibility of spillovers from other SARS-related viruses. Toward this end, biophysical and structural properties of the SARS-CoV-2 RBD and ACE2 recombinant proteins were assessed, followed by SPR-based Eyring and van’t Hoff analyses of their interaction. During these studies, it was discovered that the extent of thermal and chemical denaturation of the RBD is much greater under reducing than non-reducing conditions, implying that disulfide bonds provide structural stability to the RBD. Furthermore, the thermal unfolding of the RBD was found to be near-completely reversible under non-reducing conditions. A functional role for this feature was explored, which led to the hypothesis that disulfide bond stabilization preserves the ability to bind ACE2 following denaturation of the RBD. ACE2 binding was assessed following treatment of the RBD with high temperatures (95°C). These experiments showed that the RBD retains binding activity under non-reducing, but not reducing, conditions. These results support that disulfide bonds provide significant structural stability to the RBD and may function to preserve the RBD–ACE2 interaction in the face of environmental stress that SARS-CoV-2 may encounter during its lifecycle.

## Results

### Characterization of the SARS-CoV-2 RBD and ACE2 receptor recombinant proteins

Recombinant proteins of the SARS-CoV-2 RBD and ACE2 receptor were characterized by a variety of techniques to assess their biochemical, biophysical, and structural properties. All quantitative measures are displayed in Supplementary Table S1. Recombinant proteins were expressed in Expi293F cells, a mammalian protein expression system. Glycosylation of each protein was verified by PNGase F treatment (Figure 2A). The presence of glycans was confirmed by a mobility shift in the SDS-PAGE gel, which was exhibited by both the RBD and ACE2. PNGase F can be observed as the band that migrates to ∼28 kDa. The molecular weight of each protein was determined by MALDI-TOF (Figure 2B) and AUC sedimentation velocity (Figure 2C) analyses. For the RBD, both MALDI-TOF and AUC revealed a molecular weight of ∼32 kDa. For ACE2, the two measurements differed, reflecting the dimeric state of ACE2. MALDI-TOF detected a molecular weight of ∼109 kDa, whereas AUC measured ∼229 kDa. Due to the presence of glycans, the mass of each recombinant protein was found to be greater than the molecular weight calculated by ProtParam [82] based on its amino acid sequence. Recombinant proteins were also assessed by CD (Figure 2D). The secondary structure of the RBD was primarily composed of antiparallel β-sheets and a small percentage of α-helices. ACE2 was found to consist mostly of α-helices. These secondary structure measurements are consistent with their reported cryo-EM and X-ray crystallography structures [7,30]. Finally, nanoDSF revealed the thermal unfolding profile of each recombinant protein (Figure 2E). Both the RBD and ACE2 exhibited a two-state unfolding pattern that enabled the inflection temperature (*T*_i_) to be unambiguously estimated. The *T*_i_ values of the RBD and ACE2 were determined to be 53.0°C and 56.5°C, respectively.

**Figure 2:**
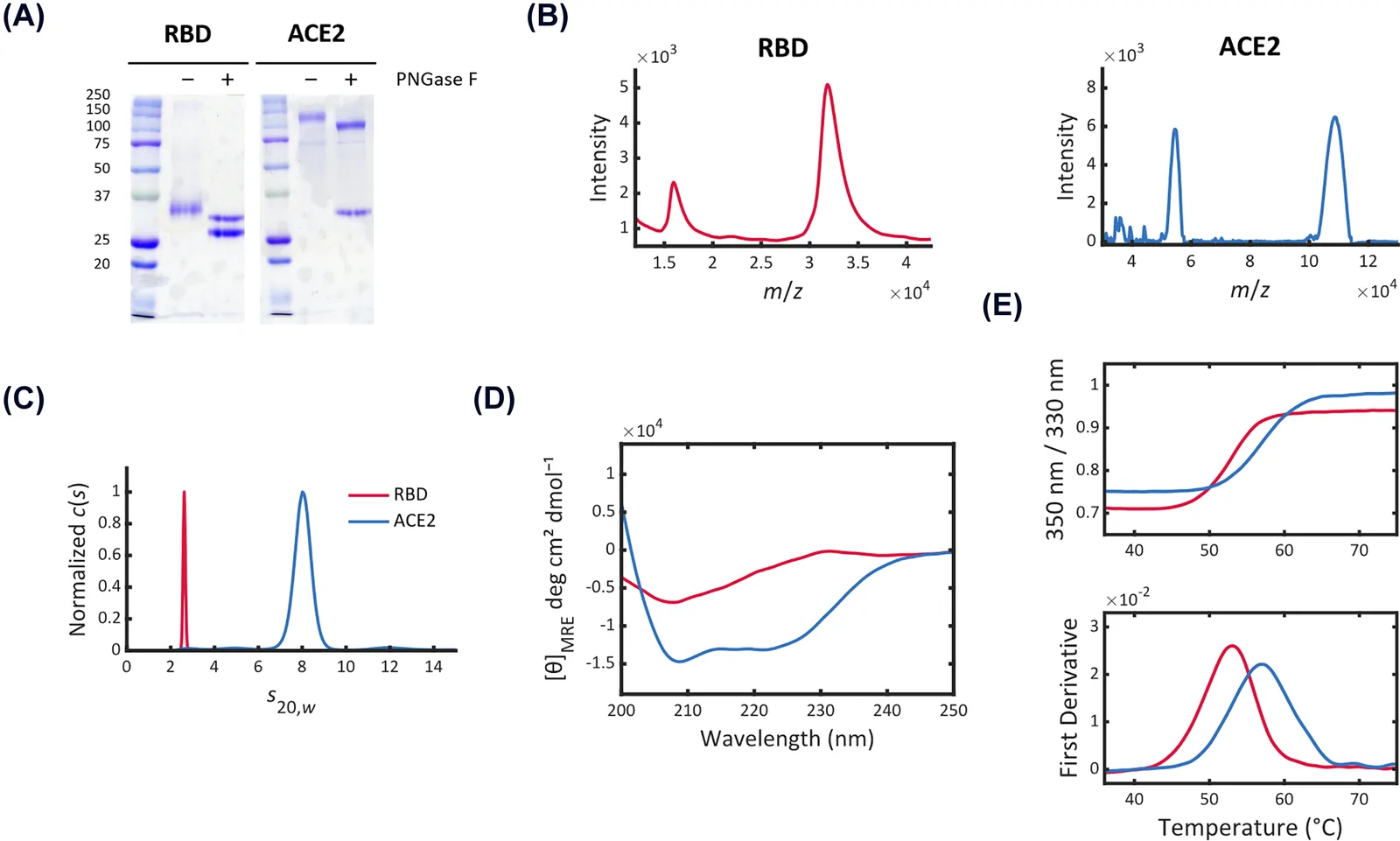
Characterization of the SARS-CoV-2 RBD (red) and ACE2 (blue) (**A**) 10% SDS–PAGE gel demonstrating a mobility shift following PNGase F treatment. Representative plots of data obtained from (**B**) MALDI-TOF, (**C**) AUC, (**D**) CD, and (**E**) nanoDSF analyses.

### Eyring and van’t Hoff analyses of the RBD–ACE2 interaction

SPR binding kinetics experiments of the RBD–ACE2 interaction were performed over a range of temperatures (Figure 3A–F). The equilibrium and rate constants obtained from these experiments are displayed in Figure 3G,H and reported in Supplementary Table S2. Decreasing temperature enhanced the affinity between the RBD and ACE2. The penalty of lowering *k*_on_ was offset by slowing *k*_off_. These data were used to construct Eyring (Figure 4A,B) and van’t Hoff (Figure 4C) plots. Changes in transition state enthalpy (Δ*H*^‡^) and entropy (Δ*S*^‡^) for binding (on) and unbinding (off) were acquired through linear regressions of the Eyring plot data. Δ*H*^‡^_on_ and Δ*S*^‡^_on_ were, respectively, determined to be 59 kJ/mol and 60 J/mol·K. These values were lower than the corresponding values of Δ*H*^‡^_off_ and Δ*S*^‡^_off_, which were measured to be 104 kJ/mol and 66 J/mol·K, respectively. The transition state Gibbs free energy changes (Δ*G*^‡^) were calculated from Δ*H*^‡^ and Δ*S*^‡^ at 25°C. Δ*G*^‡^_on_ and Δ*G*^‡^_off_ were determined to be 41 and 85 kJ/mol, respectively. The transition-state thermodynamic parameters at 25°C are displayed in Figure 4D and reported in Supplementary Table S3.

**Figure 3:**
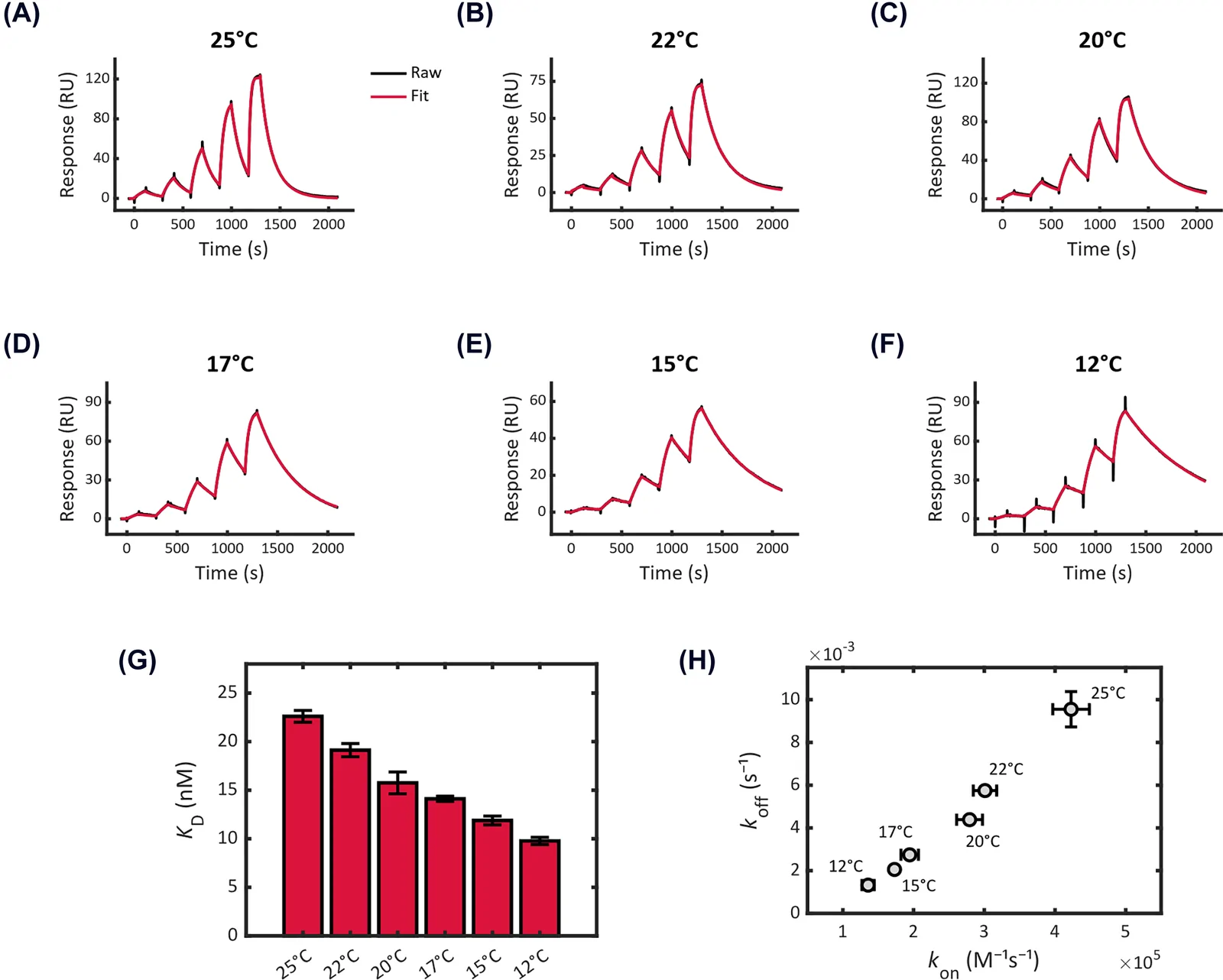
SPR binding kinetics analyses of the RBD–ACE2 interaction Representative SPR sensorgrams for experiments performed at (**A**) 25°C, (**B**) 22°C, (**C**) 20°C, (**D**) 17°C, (**E**) 15°C, and (**F**) 12°C. (**G**) Bar chart displaying the *K*_D_ determined at each temperature. (**H**) Plot of *k*_on_ and *k*_off_ determined at each temperature. Data points and error bars in panels (G,H) represent the average and standard deviation of two independent experiments.

**Figure 4:**
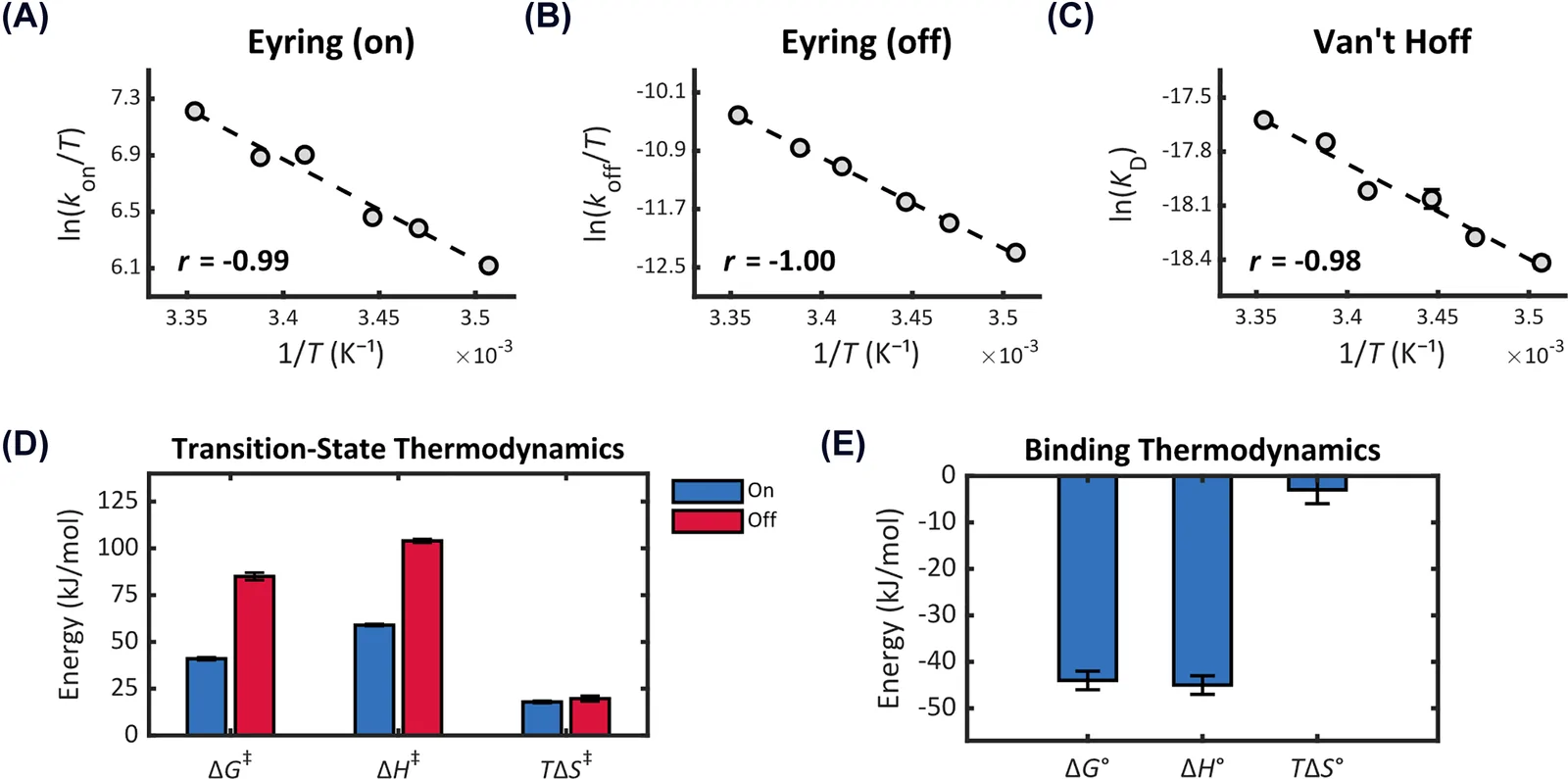
Eyring and van’t Hoff analyses of the RBD–ACE2 interaction Representative plots for the (**A**) Eyring (on), (**B**) Eyring (off), and (**C**) van’t Hoff plots. (**D**) Bar chart of transition-state thermodynamics at 25°C. (**E**) Bar chart of binding thermodynamics at 25°C. Data points and error bars in panels (A–C) represent the average and standard error of two technical replicates of a representative experiment. Data points and error bars in panels (D,E) represent the average and standard deviation of two independent experiments.

The difference between Δ*G*^‡^_on_ and Δ*G*^‡^_off_ is equivalent to the free energy difference between the bound and unbound states (Δ*G*°). This was calculated to be −44 kJ/mol, indicating that the RBD–ACE2 interaction is thermodynamically favorable (Δ*G*° < 0). Changes in the enthalpy (Δ*H*°) and entropy (Δ*S*°) were also obtained from linear regressions of the van’t Hoff plot data. They were determined to be −45 kJ/mol and −10 J/mol·K, respectively. As shown in Figure 4E, at 25°C the RBD–ACE2 interaction is driven by Δ*H*° and experiences a small penalty arising from Δ*S*°. The thermodynamic parameters for binding are also reported in Supplementary Table S4.

### Disulfide bonds stabilize the RBD and preserve ACE2 binding following thermal denaturation

While characterizing the SARS-CoV-2 RBD, we found that CD analysis provided a more complete understanding of its thermal denaturation. Figure 5A shows CD spectra acquired over a range of temperatures for the RBD. It was discovered that in the presence of the reducing agent, tris(2-carboxyethyl)phosphine (TCEP; 1 mM), the extent of RBD unfolding is much greater than under non-reducing conditions (Figure 5B). Similarly, the RBD was slightly more resistant to chemical denaturation by guanidinium (Gdn)-HCl in the absence of TCEP (Supplementary Figure S1A,B). These data indicate that disulfide bonds play a role in the stability of the RBD. Disulfide bonds within the RBD have been a topic of interest in previous reports [83–86]. However, it is not fully understood how these bonds contribute to the fitness of SARS-CoV-2.

**Figure 5:**
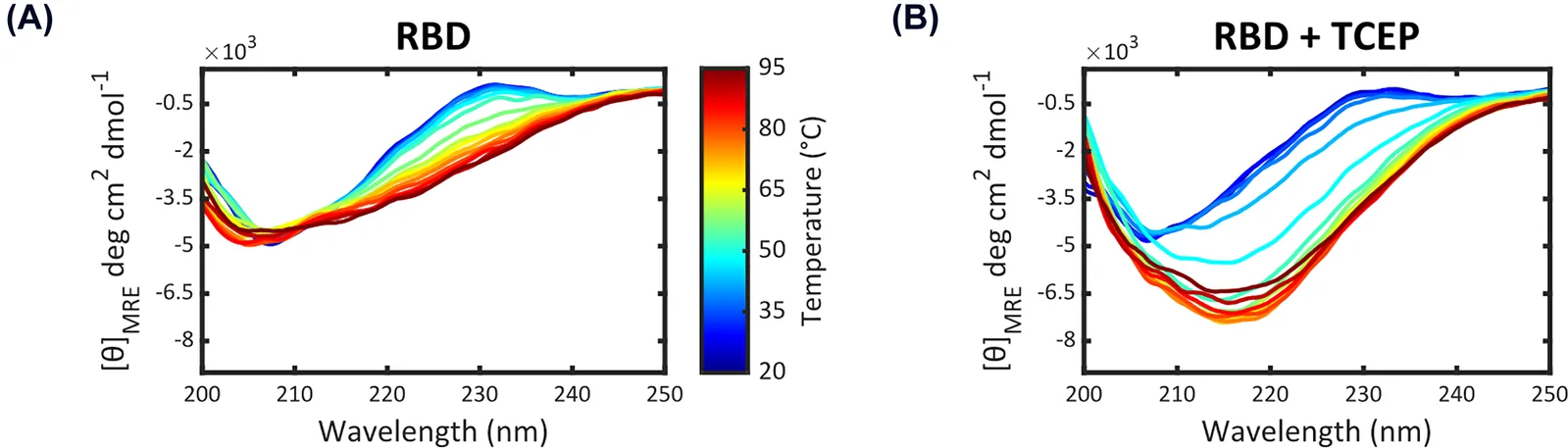
CD temperature scans of the RBD Representative plots of CD data from experiments performed in the (**A**) absence and (**B**) presence of TCEP.

The influence of disulfide bonds on the structural integrity of the RBD was further explored. The RBD was analyzed by AUC before and after heating to 95°C (with subsequent cooling to 25°C; see the ‘Materials and methods’ section) under non-reducing and reducing conditions. Remarkably, the RBD structure was unaffected by high temperatures under non-reducing conditions (Figure 6A). Its size, shape, and oligomeric state remained unchanged after heating, implying its unfolding is reversible. In the presence of TCEP, a small fraction of the RBD dimerized prior to heating. Otherwise, it retained a similar *S*-value (Figure 6B). After incubation at 95°C, the reduced RBD irreversibly unfolded, resulting in extensive aggregation. L-Arginine (50 mM) was included in the buffer that was used in most of the analyses in these studies. Since this additive is known to mitigate protein aggregation [87–89], we investigated whether the ability of the RBD to refold depended on its presence. L-Arginine was removed from the buffer, and the experiment was repeated. The RBD retained its ability to refold after thermal denaturation under non-reducing conditions (Supplementary Figure S2). However, minor aggregation of the RBD was observed after heating in the absence of L-arginine that was not detected when heated in its presence. These data support that disulfide bonds resist denaturation of the RBD and mediate its refolding. This property is further enhanced by L-arginine.

**Figure 6:**
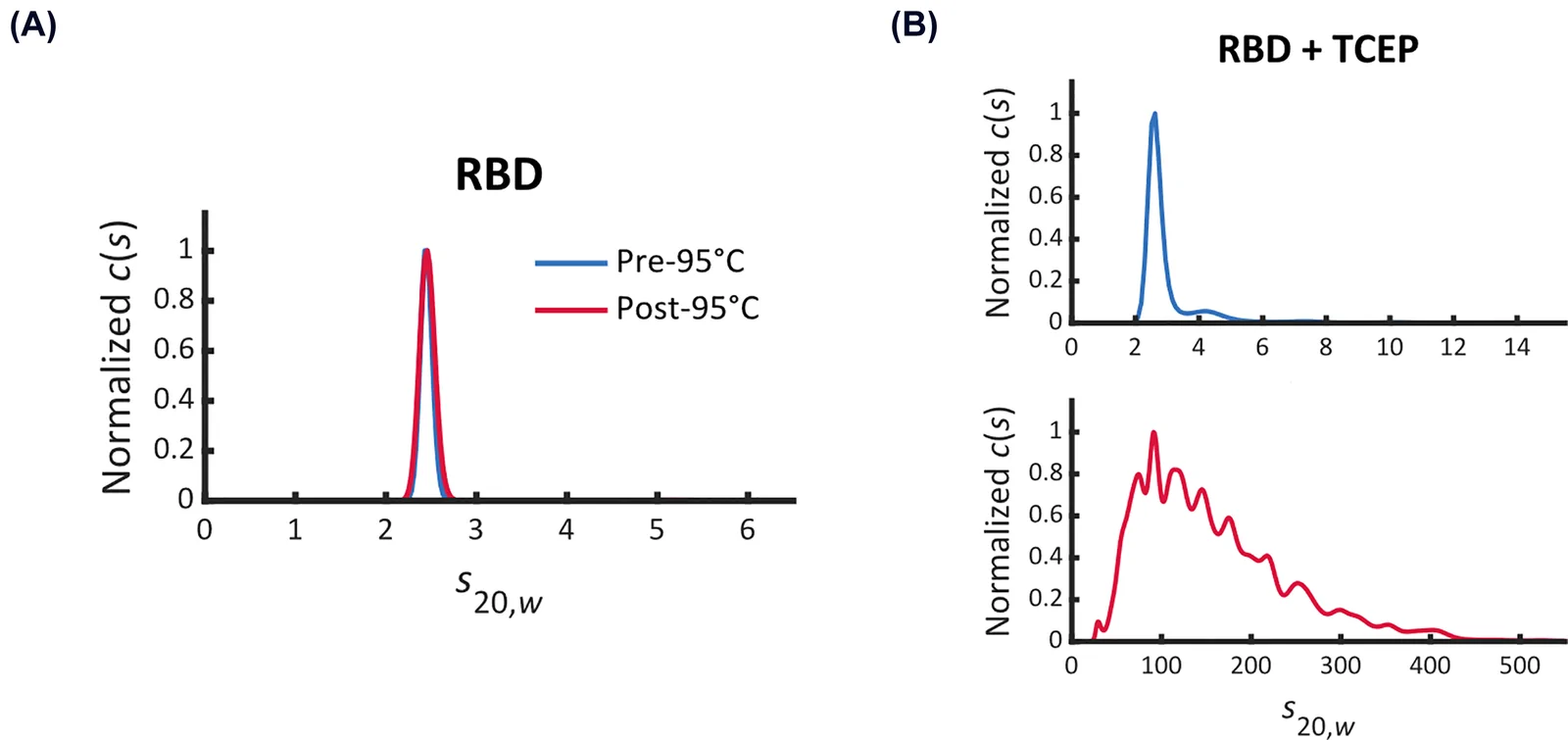
AUC analysis of the RBD before (blue) and after (red) heating to 95°C Representative plots of AUC data from experiments performed in the (**A**) absence and (**B**) presence of TCEP.

Next, a functional role that could be relevant to the fitness of SARS-CoV-2 was investigated for the observed disulfide bond-mediated stabilization of the RBD. It was hypothesized that the stability provided by these bonds would preserve the RBD structure following exposure to denaturants so that its interaction with ACE2 can be preserved. Such a feature could reflect a broader mechanism by which SARS-CoV-2 protects its receptor-binding capability from environmental stress that the virus may encounter during its lifecycle. ACE2 binding was assessed by SPR before and after heating the RBD to 95°C under non-reducing and reducing conditions. Strikingly, under non-reducing conditions and in the absence of L-arginine, the RBD retained ∼40% of its ACE2-binding activity after exposure to high temperatures (Figure 7A,D). When the RBD was heated following TCEP reduction, ACE2 binding was completely lost (Figure 7B,D). Receptor binding was also examined in the presence of L-arginine. Its addition improved ACE2-binding levels under non-reducing conditions following thermal denaturation of the RBD (Figure 7C,D). This is consistent with the ability of L-arginine to promote RBD refolding (Figure 6A and Supplementary Figure S2).

**Figure 7:**
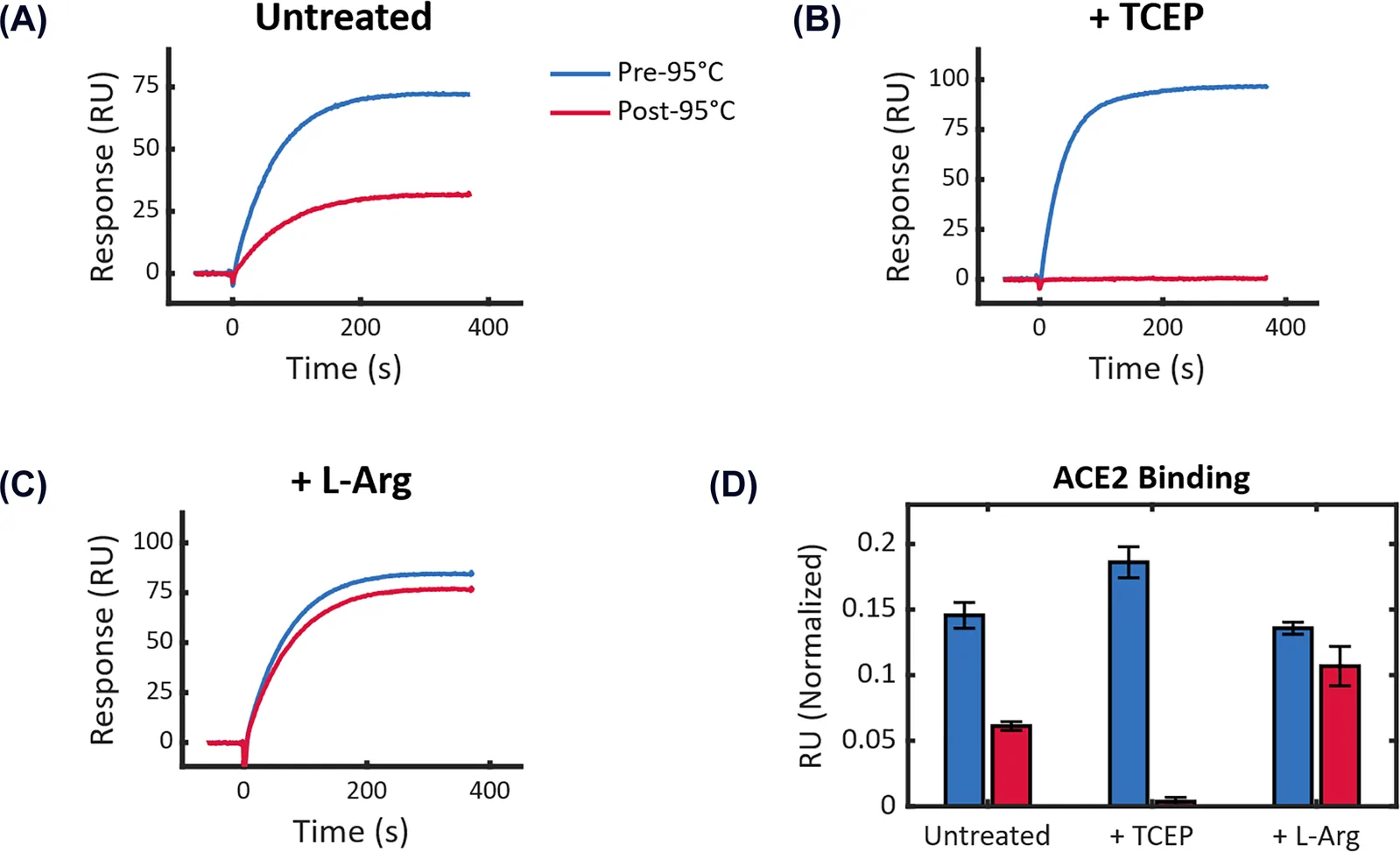
SPR binding analyses of the RBD–ACE2 interaction before (blue) and after (red) heating to 95°C Representative SPR sensorgrams for experiments performed with (**A**) untreated RBD, (**B**) RBD in the presence of TCEP (1 mM), and (**C**) RBD in the presence of L-arginine (50 mM). (**D**) Bar chart of average response (RU) recorded at 370 s normalized to the ACE2 capture level. The data points and error bars in panel (D) represent the average and standard deviation of three independent experiments.

## Discussion

The binding between the Spike protein and its receptor, ACE2, is a critical point in the cell entry mechanism of SARS-CoV-2. Therefore, a comprehensive analysis of the RBD–ACE2 interaction is vital to understanding this process. To further this understanding, recombinant proteins of the RBD and ACE2 were characterized along with their binding kinetics and thermodynamics. The glycosylation of each protein was verified by PNGase F treatment (Figure 2A). Analyses by MALDI-TOF (Figure 2B), AUC (Figure 2C), CD (Figure 2D), and nanoDSF (Figure 2E) confirmed that each protein was properly folded, in the correct oligomeric state, and monodisperse in solution (Supplementary Table S1). Such high-quality proteins are required for rigorous experimentation. The interaction between the RBD and ACE2 was then analyzed by SPR across a range of temperatures (Figure 3A–F) to perform Eyring (Figure 4A,B) and van’t Hoff (Figure 4C) analyses. The SARS-CoV-2 RBD was found to have a high affinity for its receptor (Supplementary Table S2). A slow *k*_off_ strongly contributed to this tight binding, reflecting a long residence time between Spike and ACE2. This implies that SARS-CoV-2 is capable of forming a very stable attachment to the cell surface of its human host. Eyring and van’t Hoff analyses revealed that the binding between the RBD and ACE2 is spontaneous and enthalpy driven (Figure 4D,E), which is typical of many protein–protein interactions.

While characterizing the recombinant proteins, it was discovered that disulfide bonds confer significant structural stability to the RBD. CD temperature scans revealed that in the presence of TCEP the RBD exhibits a thermal unfolding profile that is unlike the pattern observed under non-reducing conditions (Figure 5A,B). Disulfide bond reduction enhances the sensitivity of the RBD structure to increasing temperature, leading to a higher degree of unfolding. Additionally, the RBD was more resistant to chemical denaturation by Gdn-HCl under non-reducing conditions (Supplementary Figure S1A,B). AUC experiments demonstrated that disulfide bonds within the RBD enable thermal denaturation to be reversible. After heating to 95°C under non-reducing conditions, the RBD retained an *S*-value similar to that observed prior to heating (Figure 6A). L-Arginine was found to enhance this property (Supplementary Figure S2). In the presence of TCEP, thermal denaturation results in unfolding and aggregation of the RBD (Figure 6B). A functional role for these disulfide bonds and the stability that they provide was further explored. It was hypothesized that the purpose of these bonds is to preserve the receptor-binding ability of the RBD in the face of denaturants. Under non-reducing conditions, the RBD retained ∼40% of its ACE2-binding activity following thermal denaturation at 95°C (Figure 7A,D). Consistent with its ability to mitigate RBD aggregation, L-arginine recovered binding to a higher level (Figure 7C,D). In the presence of TCEP, the RBD lost the ability to bind ACE2 after treatment with high temperatures (Figure 7B,D).

The SARS-CoV-2 RBD contains four disulfide bond pairs: C336–C361, C379–C432, C391–C525, and C480–C488. Previous studies on the role of these bonds have been reported. Grishin et al. [83] performed molecular dynamics simulations of the RBD along with *in vitro* experiments that probed the effect of reducing agents on the RBD structure, ACE2-binding capability, and viral infectivity. Their simulations found that the flexibility of the RBD structure increases upon reduction of all disulfide bonds. Similar to the findings in this manuscript, they also reported that the CD spectrum of the RBD is more sensitive to increasing temperature in the presence of reducing agents. At 37°C, the CD spectrum of the RBD significantly deviated from that recorded at room temperature when incubated with DTT or TCEP, implying thermal unfolding of the RBD. Consistent with this, these investigators found that ACE2 binding was impaired by disulfide bond reduction at this temperature. This is likely a consequence of the flexibility that arose from reducing C480–C488. Similar findings have been reported in another molecular dynamics study [84]. In the studies presented here, it was found that TCEP reduction mildly improved binding to ACE2 (Figure 7A,B,D). This is likely due to the difference in temperatures at which these experiments were performed. The structural instability of the RBD under reducing conditions was shown to emerge at physiological temperatures (Figure 5A,B). The SPR binding experiments reported here were performed at 25°C. Grishin et al. [83] also demonstrated that reducing agents inhibit viral replication of SARS-CoV-2 in cell culture. Similar reports [85,86] have shown that reducing agents attenuate replication of SARS-CoV-2 along with the fusogenicity of the Spike protein.

The data presented here offer a new perspective on the functional role of disulfide bonds within the RBD. As discussed above, previous reports [83–86] suggest that these bonds are required for the interaction between the Spike protein and ACE2. This stable attachment then enables the entry of SARS-CoV-2 into host cells. While this may be true, it is also possible that the structural stability provided by disulfide bonds belongs to a broader mechanism that protects the RBD from environmental stress in an effort to preserve the RBD–ACE2 interaction. During the SARS-CoV-2 lifecycle, the virus will encounter a variety of denaturants from inside and outside the host. These may include rapid temperature fluctuations, UV radiation damage, oxidative stress, and harmful pH conditions. Here, it was demonstrated that the RBD can retain its ACE2-binding ability after being heated to temperatures as high as 95°C. While such temperatures would certainly be detrimental to the structural integrity of the whole SARS-CoV-2 virion, this feature highlights the extent to which disulfide bonds protect the RBD and the importance of preserving its interaction with ACE2.

Although the findings in the present study suggest a role for disulfide bond-mediated RBD stabilization in the fitness of SARS-CoV-2, direct evidence remains to be established and requires experiments with the authentic SARS-CoV-2 virus. Additionally, it is unclear whether this feature is a result of the collective action of all four disulfide bond pairs or relies on a select few. Insights into these questions can be provided from aligning RBD sequences from various coronavirus lineages to observe how evolutionarily conserved their cysteine residues are. As shown in Supplementary Figure S3, these disulfide bonds are broadly conserved among several coronaviruses, indicating their evolutionary importance, with the exception of C480–C488 (relative to SARS-CoV-2). This disulfide bond pair is located within the RBM near the RBD–ACE2 interface. Coronaviruses Rc-o319, Rc-kw8, and Rp3 each feature a deletion in the RBM sequence that results in the loss of the participating cysteine residues. A recent study examining the adaptiveness of multi-species ACE2 usage found that RBD variants bearing this deletion were associated with a narrow ACE2 ortholog tropism and were unable to bind human ACE2 [48]. MERS-CoV also lacks C480–C488 but features C503–C526 in a nearby region. Given their proximity, these two disulfide bond pairs may play comparable roles in their respective backgrounds. Their differing positions may reflect adaptation to distinct receptors, ACE2 [30] and DPP4 [90]. Disulfide bonds within the RBM sequence likely play a role in host receptor binding and recognition. Disulfide bonds outside the RBM may be the source of the structural stability provided to the RBD. Mutagenesis experiments that disrupt single disulfide bond pairs and evaluate their structural impact could clarify this. Beyond SARS-CoV-2, disulfide bonds within the RBDs of other coronaviruses should be examined to determine if this property is conserved. Such studies would be informative about the relevance of disulfide bond-mediated RBD stabilization to the fitness and evolution of coronaviruses.

## Materials and methods

### DNA materials

The RBD (residues 331–528) DNA sequence was PCR amplified from SARS-CoV-2 S HexaPro (Addgene #154754; a gift from Jason McLellan [91]) and cloned into a vector backbone that featured an N-terminal μ-phosphatase signal peptide and C-terminal V5- and 8× His tags. The ACE2 ectodomain (residues 18–740) DNA sequence was PCR amplified from pcDNA3.1-hACE2 (Addgene #145033; a gift from Fang Li [92]) and cloned into a vector backbone that featured an N-terminal μ-phosphatase signal peptide and C-terminal V5-, SBP, and 8× His tags. Each DNA vector was prepared for transfections using the EndoFree Plasmid Maxi Kit (Qiagen). All DNA vector backbones were synthesized by Twist Bioscience. The primer sequences (5′–3′) for PCR are as follows: RBD Fwd (tctagaaacattaccaacctctgcccct), RBD Rev (ccatggcttaggaccgcagacggtgg), ACE2 Fwd (tctagacagtccaccattgaggaacagg), and ACE2 Rev (accggtggaaacagggggctgg).

### Recombinant protein expression and purification

All recombinant proteins used in these studies were expressed using the Expi293 protein expression system (Thermo Fisher). Expi293F cell cultures were maintained and transfected as recommended by the manufacturer. Six days post-transfection, recombinant proteins were harvested from cell culture and purified over Ni-Sepharose High Performance resin (Cytiva). Recombinant RBD was further purified via gel filtration over a Bio-Gel P-60 resin (Bio-Rad). Following purification, each recombinant protein was buffer exchanged into PBS, 50 mM L-arginine HCl, pH 7.4, using a PD-10 column (Cytiva).

### MALDI-TOF

MALDI-TOF analysis of proteins was performed using an UltrafleXtreme MALDI-TOF-TOF (Bruker). Samples were diluted to 0.25 mg/ml in PBS, mixed at a 1:9 ratio with sinapinic acid, and spotted on a MALDI target plate. Data were acquired using flexControl (Bruker) and analyzed with flexAnalysis (Bruker) software. Spectra were smoothed using a Savitzky-Golay filter with a 2000 m/z width. MALDI-TOF data presented in Supplementary Table S1 represent the average and standard deviation of three independent analyses.

### AUC

AUC analysis of proteins was performed using an Optima XL-I (Beckman Coulter) analytical ultracentrifuge with an An-60 Ti rotor (Beckman Coulter). Unless stated otherwise, the samples were diluted with PBS, 50 mM L-arginine HCl, pH 7.4, to a concentration such that the measured *A*_280_ was 0.25. AUC cells with double-sector centerpieces and quartz windows were used to house the samples. All experiments were performed at 20°C and 30,000 rpm with the exception of those conducted on the RBD after heating to 95°C under reducing conditions, which were performed at 10,000 rpm. The Optima XL-I ultracentrifuge was operated using ProteomeLab (Beckman). AUC data were analyzed using SEDFIT [93]. Partial specific volumes, buffer density, and buffer viscosity were calculated using SEDNTERP3 [94]. The AUC data presented in Supplementary Table S1 represent the average and standard deviation of three independent analyses. AUC experiments with the RBD under non-reducing and reducing conditions before and after heating to 95°C were performed in two independent replicates. The RBD was heated in a thermocycler from 25°C to 95°C in 2.5°C increments and 30 s steps at the concentration used in AUC analysis (5.33 μM), followed by cooling from 95°C to 25°C in the same manner.

### CD

CD analyses of proteins were performed using a J-1500 CD spectrophotometer (Jasco). Protein samples were buffer exchanged into 20 mM Na_2_HPO_4_, pH 7.4, and diluted to a final concentration of 0.15 mg/ml. For each sample, CD spectra were collected over 3 accumulations at 20°C, 195–250 nm, 50 nm/min scanning speed, and a 2 s digital integration time. Each measurement was baseline corrected to the buffer background. Temperature scans were performed under the same conditions from 20°C to 95°C with data collected every 5°C. For TCEP-treated samples, protein was incubated in 1 mM TCEP for 30 min prior to heating. For Gdn-HCl-treated samples, protein was incubated in 2 M Gdn-HCl for 30 min. All data were exported for smoothing (moving average window = 25) and plotting in MATLAB. Secondary structure analysis of CD spectra was performed with BeStSel [95,96]. CD data presented in Supplementary Table S1 represent the average and standard deviation of two independent analyses. All other CD experiments were performed in two independent replicates.

### NanoDSF

NanoDSF analyses of proteins were performed using a TychoNT.6 (NanoTemper). Each sample was diluted to 0.25 mg/ml in PBS, 50 mM L-arginine HCl, pH 7.4, before analysis. The melting point determined by the instrument is reported as the inflection temperature (*T*_i_). NanoDSF data presented in Supplementary Table S1 represent the average and standard deviation of three independent analyses.

### SPR

SPR analyses of the RBD–ACE2 interaction were performed using a Biacore X100 (Cytiva). For each experiment, streptavidin (0.45 mg/ml diluted in Acetate 4.5) was immobilized on a CM5 SPR chip (Cytiva) to an RU level of 3000–5000 via amine coupling. ACE2 (∼100 μg/ml) was captured through its C-terminal SBP-tag to properly orient it for binding. PBST (0.005% Tween-20), 50 mM L-arginine HCl, pH 7.4, was used as the running buffer for each experiment. Single-cycle kinetic analyses were performed to obtain data used in the Eyring and van’t Hoff plots. The RBD was sequentially injected at concentrations of 2, 6, 18, 54, and 162 nM with 120 s injection times and a final 800 s dissociation time. Glycine 1.5 (Cytiva) was injected between each cycle to regenerate the surface of the chip. All traces were double referenced to buffer injections. The Biacore evaluation software (Cytiva) was used to determine *K*_D_, *k*_on_, and *k*_off_ values. The following equations were used for the Eyring (eqn 1) and van’t Hoff (eqn 2) analyses:

(1) $$\ln\left(\frac{k}{T}\right)=\frac{-\Delta H^{‡}}{RT}+\ln\frac{k_{B}}{h}+\frac{-\Delta S^{‡}}{R},$$

(2) $$\ln(K_{D})=\frac{\Delta H{}^{\circ}}{RT}-\frac{\Delta S{}^{\circ}}{R},$$

where *k* = rate constant; *T* = temperature; *R* = 8.315 J·mol^-1^·K^-1^;

*k*_*B*_ = 1.380 × 10^−23^ m^2^·kg ·s^−2^ · *K*^-1^; *h* = 6.626 × 10^−34^ m^2^·kg·*s*^−1^.

Linear regressions of the data used in the Eyring and van’t Hoff plots were performed in MATLAB. For experiments evaluating the ACE2-binding activity of the RBD following thermal denaturation, the RBD was heated in a thermocycler from 25°C to 95°C in 2.5°C increments and 30 s steps at a concentration of 1 μM, followed by cooling from 95°C to 25°C in the same manner. For TCEP-treated samples, the RBD was incubated in 1 mM TCEP for 30 min prior to heating. ACE2 binding was then assessed by injecting the RBD at a concentration of 25 nM for 370 s.

### Sequence alignment

RBD sequences were obtained from GenBank (SARS-CoV-2: MN908947.3; SARS-CoV: NC_004718.3; RaTG13: MN996532.2; WIV1: KF367457.1; Rc-o319: BCG66627.1; Rc-kw8: BDD37176.1; Rp3: AAZ670 52.1; Khosta-2: MZ190138.1; PCoV_GX-P2V: MT072864.1; MERS-CoV: KC164505.2), aligned using ClustalX2 [97], and visualized with Jalview [98].

## Supplementary Material

Supplementary Figures S1-S3 amd Tables S1-S4

## Data Availability

Data will be made available upon reasonable request to the corresponding author.

## Abbreviations

ACE2: angiotensin-converting enzyme 2
AUC: analytical ultracentrifugation
CD: circular dichroism
DSF: differential scanning fluorimetry
Gdn: guanidinium
MALDI-TOF: matrix-assisted laser desorption/ionization time-of-flight
RBD: receptor-binding domain
RBM: receptor-binding motif
SARS-CoV-2: severe acute respiratory syndrome coronavirus 2
SPR: surface plasmon resonance
TCEP: tris(2-carboxyethyl)phosphine
