# Supplementary material for: Disulfide bonds stabilize the SARS-CoV-2 RBD and preserve binding to ACE2 following thermal denaturation
Source: Biosci Rep. 2026 Sep 29;46(10):BSR20260440. doi: 10.1042/BSR20260440 (PMC13623820; doi:10.1042/BSR20260440)
Supplement: Supplementary file 1 [file BSR-2026-0440-T_supp.pdf]

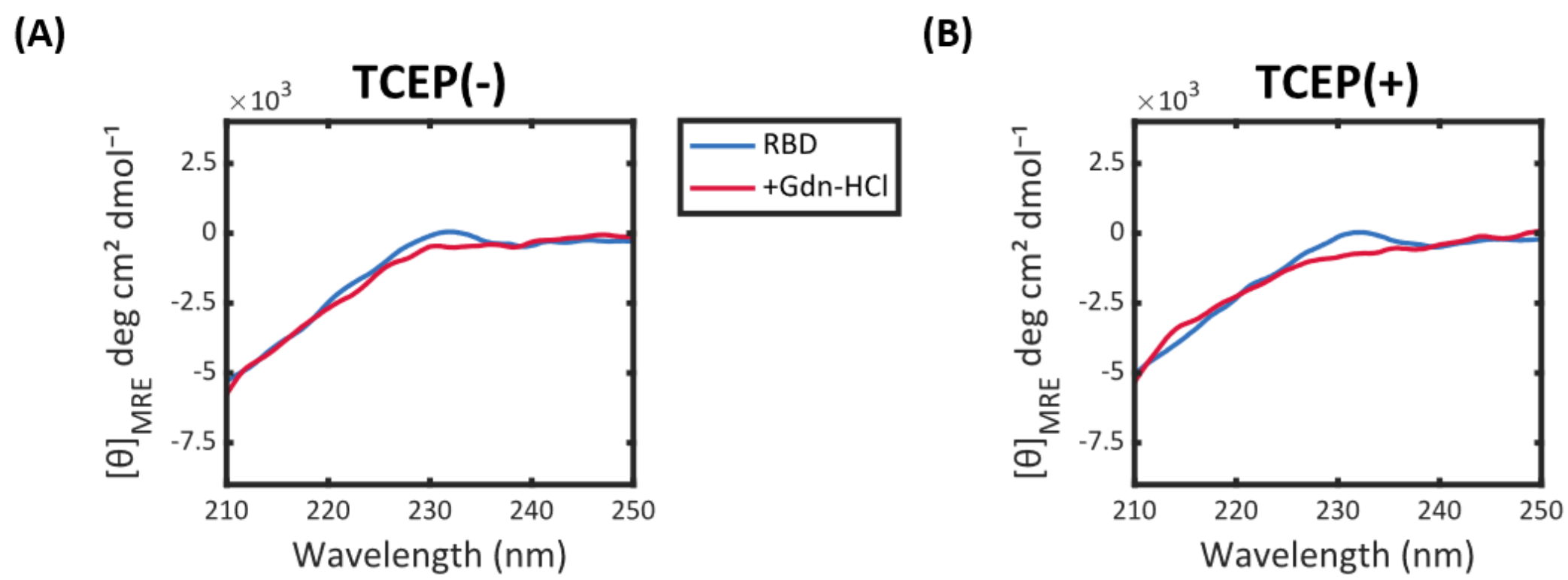

Figure S1 CD analysis of the RBD in the (A) absence and (B) presence of TCEP before (blue) and after (red) treatment with 2 M Gdn-HCl.

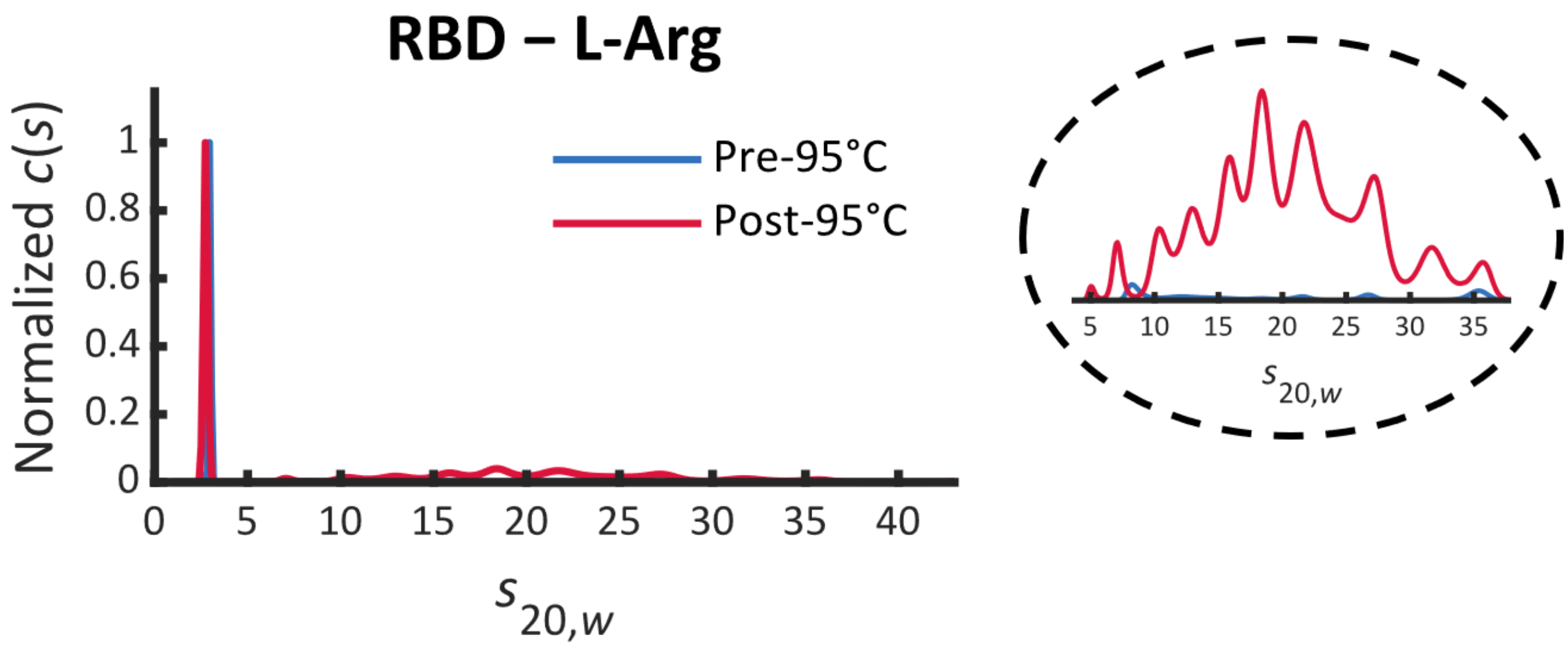

Figure S2 AUC analysis of the RBD before (blue) and after (red) heating to 95°C in the absence of L-Arginine.

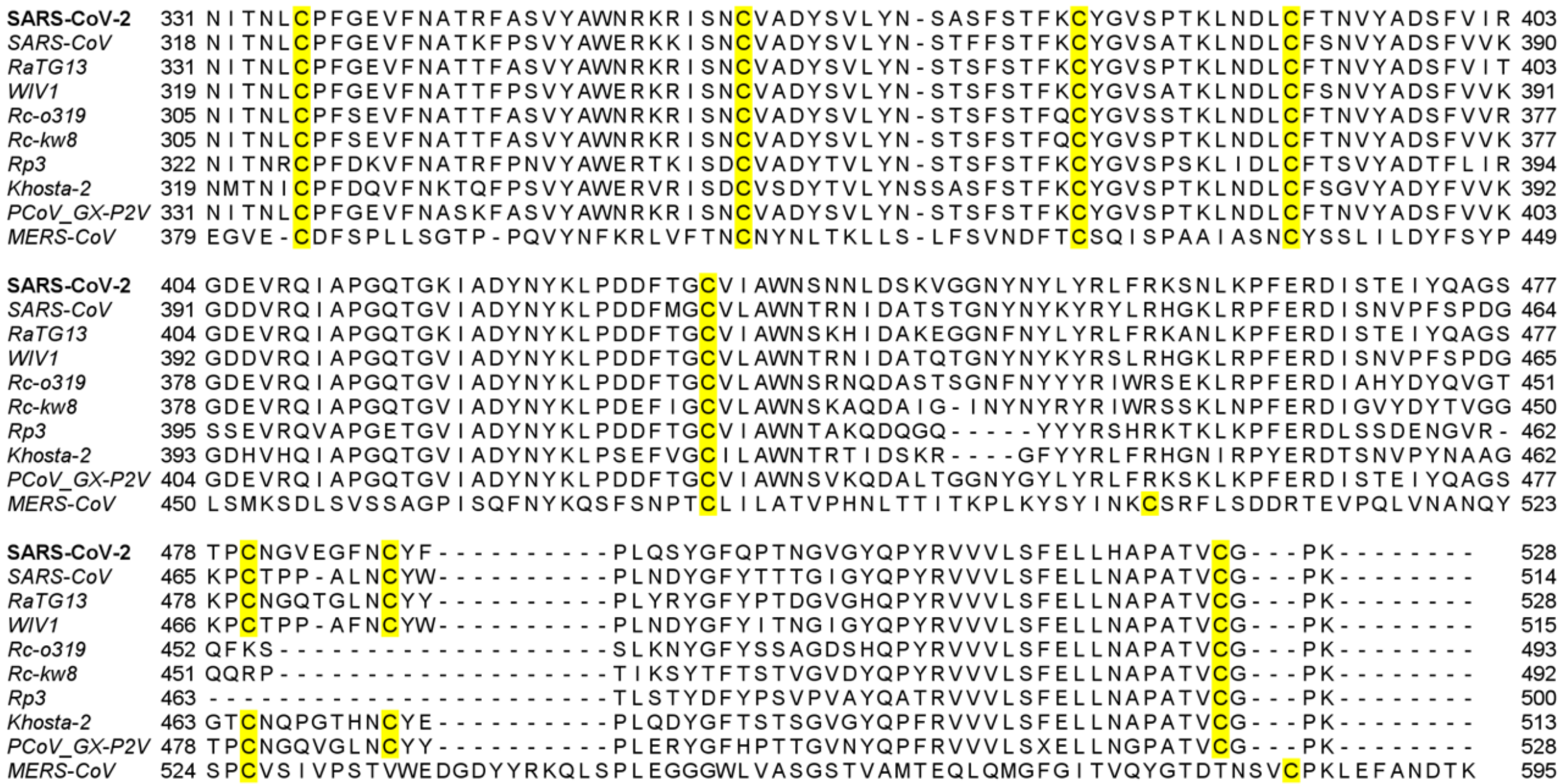

Figure S3 Alignment of coronavirus RBD sequences. Cysteine residues (C) highlighted in yellow.

Table S1 Measurements from SARS-CoV-2 RBD and ACE2 analyses.

	**RBD**	**ACE2**
Predicted MW (Da)	27644	92213
MALDI-TOF (m/z)	31790 ± 40	108700 ± 200
AUC (kDa)	32.3 ± 0.1	229 ± 8
$s_{20,w}$	2.618 ± 0.004	8.02 ± 0.02
f_r	1.457 ± 0.004	1.71 ± 0.04
α-helix (%)	6 ± 2	37 ± 1
Antiparallel β-sheet (%)	29 ± 5	5 ± 6
Parallel β-sheet (%)	0 ± 0	4.2 ± 0.1
Turn (%)	14 ± 3	12 ± 0.6
Other (%)	50.6 ± 0.5	42 ± 8
T_{i} (°C)	53.0 ± 0.1	56.5 ± 0.3

Table S2 RBD–ACE2 binding rate and equilibrium constants.

	**25°C**	**22°C**	**20°C**	**17°C**	**15°C**	**12°C**
k_{on} ($M^{-1}s^{-1}$)	4.2 ± 0.3 × 10^5	3.0 ± 0.1 × 10^5	2.8 ± 0.2 × 10^5	1.9 ± 0.1 × 10^5	1.73 ± 0.03 × 10^5	1.36 ± 0.09 × 10^5
k_{off} (s^{-1})	9.5 ± 0.8 × 10^{-3}	5.7 ± 0.1 × 10^{-3}	4.38 ± 0.03 × 10^{-3}	2.7 ± 0.1 × 10^{-3}	2.1 ± 0.1 × 10^{-3}	1.32 ± 0.03 × 10^{-3}
K_{D} (nM)	22.6 ± 0.6	19.1 ± 0.7	16 ± 1	14.1 ± 0.3	11.8 ± 0.5	9.8 ± 0.5

Table S3 RBD–ACE2 transition state thermodynamics (25°C)

	$\Delta G^{\ddagger}$ (kJ/mol)	$\Delta H^{\ddagger}$ (kJ/mol)	$T\Delta S^{\ddagger}$ (kJ/mol)	$\Delta S^{\ddagger}$ (J/mol·K)
Binding (on)	41.0 ± 0.8	59.0 ± 0.6	17.9 ± 0.6	60 ± 2
Unbinding (off)	85 ± 2	104 ± 1	20 ± 1	66 ± 5

Table S4 RBD–ACE2 binding thermodynamics (25°C).

ΔG° (kJ/mol)	ΔH° (kJ/mol)	$T\Delta S^{\circ}$ (kJ/mol)	ΔS° (J/mol·K)
−44 ± 2	−45 ± 2	−3 ± 3	−10 ± 10
